# A shared numerical magnitude representation evidenced by the distance effect in frequency-tagging EEG

**DOI:** 10.1038/s41598-022-18811-7

**Published:** 2022-08-26

**Authors:** Cathy Marlair, Virginie Crollen, Aliette Lochy

**Affiliations:** 1 Institute of Psychology (IPSY) and Institute of Neuroscience (IoNS), Université Catholique de Louvain, Place Cardinal Mercier 10, 1348 Louvain-la-Neuve, Belgium; 2 Department of Behavioral and Cognitive Sciences, Faculty of Humanities, Social and Educational Sciences, Institute of Cognitive Science and Assessment, Université du Luxembourg, Esch-sur-Alzette, Luxembourg

**Keywords:** Cognitive neuroscience, Human behaviour

## Abstract

Humans can effortlessly abstract numerical information from various codes and contexts. However, whether the access to the underlying magnitude information relies on common or distinct brain representations remains highly debated. Here, we recorded electrophysiological responses to periodic variation of numerosity (every five items) occurring in rapid streams of numbers presented at 6 Hz in randomly varying codes—Arabic digits, number words, canonical dot patterns and finger configurations. Results demonstrated that numerical information was abstracted and generalized over the different representation codes by revealing clear discrimination responses (at 1.2 Hz) of the deviant numerosity from the base numerosity, recorded over parieto-occipital electrodes. Crucially, and supporting the claim that discrimination responses reflected magnitude processing, the presentation of a deviant numerosity distant from the base (e.g., base “2” and deviant “8”) elicited larger right-hemispheric responses than the presentation of a close deviant numerosity (e.g., base “2” and deviant “3”). This finding nicely represents the neural signature of the distance effect, an interpretation further reinforced by the clear correlation with individuals’ behavioral performance in an independent numerical comparison task. Our results therefore provide for the first time unambiguously a reliable and specific neural marker of a magnitude representation that is shared among several numerical codes.

## Introduction

Humans must everyday deal with numerical information presented under various formats, codes and contexts. Imagine for example an activist demonstrating for the environment’s sake who is asked to quickly estimate and then communicate the number of people gathered for the event. The activist’s capacity to rapidly estimate the number of people will, on the one hand, rely on the so-called *Approximate Number System* (ANS), a preverbal process supporting the ability to grasp the numerosity of non-symbolic quantities (e.g., collections of items)^1,2^. Its ability to share this numerical information to others will, on the other hand, depend on an acquired *Symbolic Number System* (SNS) based on specific, learned codes (e.g., Arabic digits or number words)^2–4^. The mapping between the ANS and the SNS, which is considered as an essential step for the development of mathematical thinking^5–7^, is furthermore supported by *iconic* representations of quantities^8,9^, such as canonical finger configurations (i.e., typical configurations of the hands used by an individual to count or to show a specific number) or canonical dot patterns (i.e., patterns found on play cards or dices).

A major issue in numerical cognition investigated here, is to understand how these various formats used to represent numerosities are abstracted and integrated in order to access the underlying common magnitude information. At first sight, the three formats are characterized by very different surface features leading to an increasing precision in the representation of numerosity, from the approximate non-symbolic, to the intermediate iconic and finally to the very precise symbolic format. However, some evidence suggests that they may be integrated in one shared magnitude representation system. First, a *distance effect* is found in both symbolic and non-symbolic comparison tasks [^10–12^, but see Ref.^13^]. This effect refers to the improvement in our capacity to discriminate two numbers as the numerical distance between them increases (e.g., 3 versus 9 is easier than 3 versus 4)^14,15^. For symbolic numbers, given that any pair of symbols (e.g., 2/9 or 2/6) should be as discriminable, this finding suggests that they automatically activate their corresponding non-symbolic representation [^16^, but see Ref.^17^]. Second, neuroimaging studies have consistently indicated a *common brain region* within the parietal cortex, lying in bilateral intraparietal sulci (IPS), to support the representation of magnitude irrespectively of the numerosity format^18–22^, and this, in various tasks involving numerical comparisons. Finally, the brain activation level in bilateral IPS was found to be *modulated by the distance* between the numbers to be compared^22–25^. Thus, available evidence suggests the existence of a shared magnitude representation system, adequately assessed by the distance effect, a reliable marker of numerical semantic access.

However, the evaluation of magnitude processing in explicit comparison tasks could be affected by non-numerical factors like the available perceptual visual information (e.g., size, length, density, similarity, etc.), especially for the non-symbolic format^26^. To overcome this issue, a new line of research has focused on the use of an implicit frequency-tagging EEG paradigm called the *fast periodic visual stimulation* (FPVS) design. This approach is based on the steady-state visual evoked potentials: under periodic visual stimulation at a certain frequency, brain regions coding for this visual input elicit a response at the exact same frequency. Therefore, knowing the stimulation frequency, it is possible to track the brain response with electroencephalography^27^. Frequencies of interest usually consist in a higher rate, reflecting the general visual stimulation (e.g., 6 images/s = 6 Hz) and a lower rate, at which a deviant stimulus is periodically inserted within the stream of images (e.g., every fifth image is a deviant, so that deviants appear at 6 Hz/5 = 1.2 Hz). Analyzing the brain responses elicited by the stimulation, a low-level response synchronized with the general (higher rate) stimulation frequency should emerge and, if the brain is able to both discriminate every deviant stimulus and generalize over the deviants’ category, a selective response should also be observed at the lower rate frequency of the deviant presentation^27^. Recently, this paradigm was shown to provide a reliable measure of the spontaneous and automatic processing of non-symbolic^28–30^ and symbolic^31^ numerosities. In the latter study^31^, a stream of *base* digits were presented at a constant frequency of 10 Hz and a periodic *deviant* digit was introduced every eight stimuli (deviant presentation frequency of 10 Hz/8 = 1.25 Hz). To examine *magnitude* processing, digits smaller and larger than five were contrasted as base/deviant (and vice versa). In a *control* condition, the digits were arbitrary allocated to the base/deviant categories. Significant brain responses synchronized with the deviant magnitude presentation (1.25 Hz) were recorded over occipito-parietal regions, suggesting automatic discrimination of a magnitude change (smaller/larger than 5). However, and more unexpectedly, some responses were also observed in the control condition, suggesting that the brain was able to create a deviant category based on temporarily constructed rules. Therefore, it cannot be excluded that associative and/or physical confounding factors may have biased the responses observed in the experimental condition. This caveat is intrinsically linked to the use of Arabic digits, as there are only 9 different elements.

One potential way to address this issue could therefore be to vary the representation codes of numerosities. Increasing the variety of low-level physical parameters would indeed considerably reduce the possibility for the brain to create arbitrary associations or to detect regular repetitions. This would also give the possibility to assess numerical magnitude processing at a higher level of abstraction, as it would require participants to generalize magnitude information across formats. Accordingly, a recent study^32^ used the same magnitude contrast (small/large^31^) but with mixed codes (i.e., dots and digits, number words and dots, digits and number words). Results suggest an automatic integration of magnitude across number words and dots, digits and number words, but not dots and digits. There are, however, two limitations that are worth mentioning. First, the use of the non-symbolic format (i.e., dots) seems inadequate in a design which contrasts small (< 5) and large (> 5) numerosities. Small non-symbolic numerosities (below 4) were repeatedly shown to benefit from greater precision (i.e., they can be *subitized*) and better mapping with their corresponding symbolic representation^33–37^. Numerosities smaller than 3, moreover, have almost identical non-symbolic and iconic representations, while these two formats are clearly distinct for larger numerosities. Additionally, as small symbolic numerosities are more frequently encountered in daily life, it cannot be excluded that the responses obtained^32^ actually reflect a difference of complexity in the processing of small *versus* large numerosities, rather than the processing of magnitude per se. A second limitation is that the representation codes were only compared two by two, which can supposedly be explained by the fact that the symbolic format contains two different codes (i.e., digits and number words) while the non-symbolic format is only represented by dots. While these comparisons allow for an examination of integration across formats, they only partially address the issue of low-level physical parameter biases discussed above.

**Figure 1:**
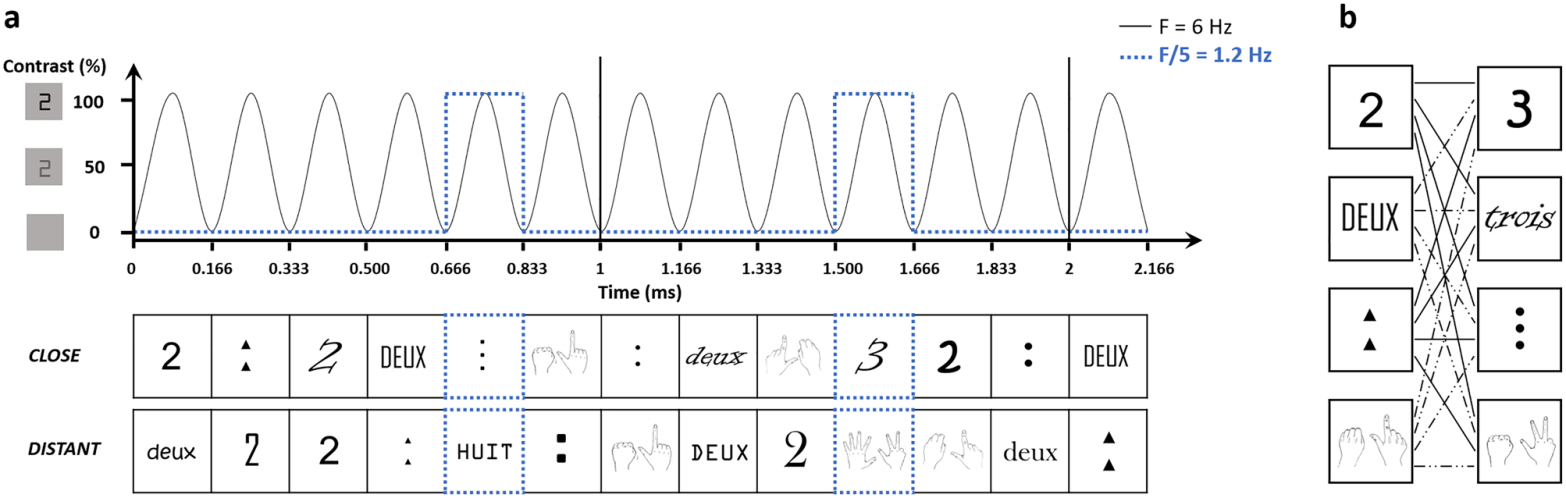
(**a**) EEG experimental design. Two numerosities were presented in randomly mixed codes (digit, word, dots, fingers) at a constant frequency of 6 Hz (i.e., 6 stimuli per second): a base numerosity (for instance “2”) and a deviant numerosity (for instance “3” in the close condition and “8” in the distant condition) which appeared every fifth base stimuli (i.e., at 6 Hz/5 = 1.2 Hz, highlighted in blue). (**b**) Behavioral task design. Participants had to choose the smaller/larger of two numerosities (for instance the close pair “2/3”) presented in every possible code (digit, word, dots, fingers). Note: “deux” means “two” in French, “trois” means “three” and “huit” means “eight”.

In the current study, we overcame these two limitations by using two symbolic codes—Arabic digits and number words—and two iconic codes—canonical dot patterns and canonical finger configurations—that were mixed in order to create a multi-code design and combined with a FPVS paradigm. Going further than previous studies, we used here a more fine-grained measure of access to number semantics—the distance effect—instead of a rather coarse small/large magnitude contrast. Participants were visually presented with a stream of one specific numerosity (the base) at a constant frequency of 6 Hz (i.e., 6 stimuli per second) and a deviant numerosity was periodically inserted within the stream every fifth stimulus (i.e., 6 Hz/5 = 1.2 Hz). The deviant numerosity could be either close (e.g., 2222/*3*/2…, distance = 1) or distant (e.g., 2222/*8*/2…, distance = 6) from the base numerosity (Fig. 1a and Supplementary MovieS1), with a total of four close pairs (i.e., 2/3, 3/2, 8/9, 9/8) and four distant pairs (i.e., 2/8, 8/2, 3/9, 9/3). Finally, both the base and deviant numerosities were presented in randomly mixed codes (i.e., digit, word, dots and fingers). In addition to the brain response synchronized with the general visual stimulation rate (6 Hz), a brain response at the deviant presentation frequency (1.2 Hz) is expected if the brain is able not only to *discriminate* the deviant from the base numerosity (e.g., 3 is different from 2) but also to *generalize* over the deviant’s various representation codes (e.g., the deviant is always 3 but presented as digit, word, dots or fingers). Furthermore, if this discrimination response truly reflects magnitude processing, a modulation of its amplitude should be observed according to the distance between the base and the deviant stimuli (e.g., larger discrimination responses in distant than close sequences). Participants were finally asked to perform a behavioral comparison task in which they had to choose the smaller/larger of two numerosities. Pairs of numerosities were the same as in the EEG experiment and also presented in randomly mixed codes (Fig. 1b). The observation of a correlation between EEG and behavioral data would support the assumption that our FPVS multi-code design provides a reliable measure of magnitude processing.

## Results

### EEG responses

**Figure 2:**
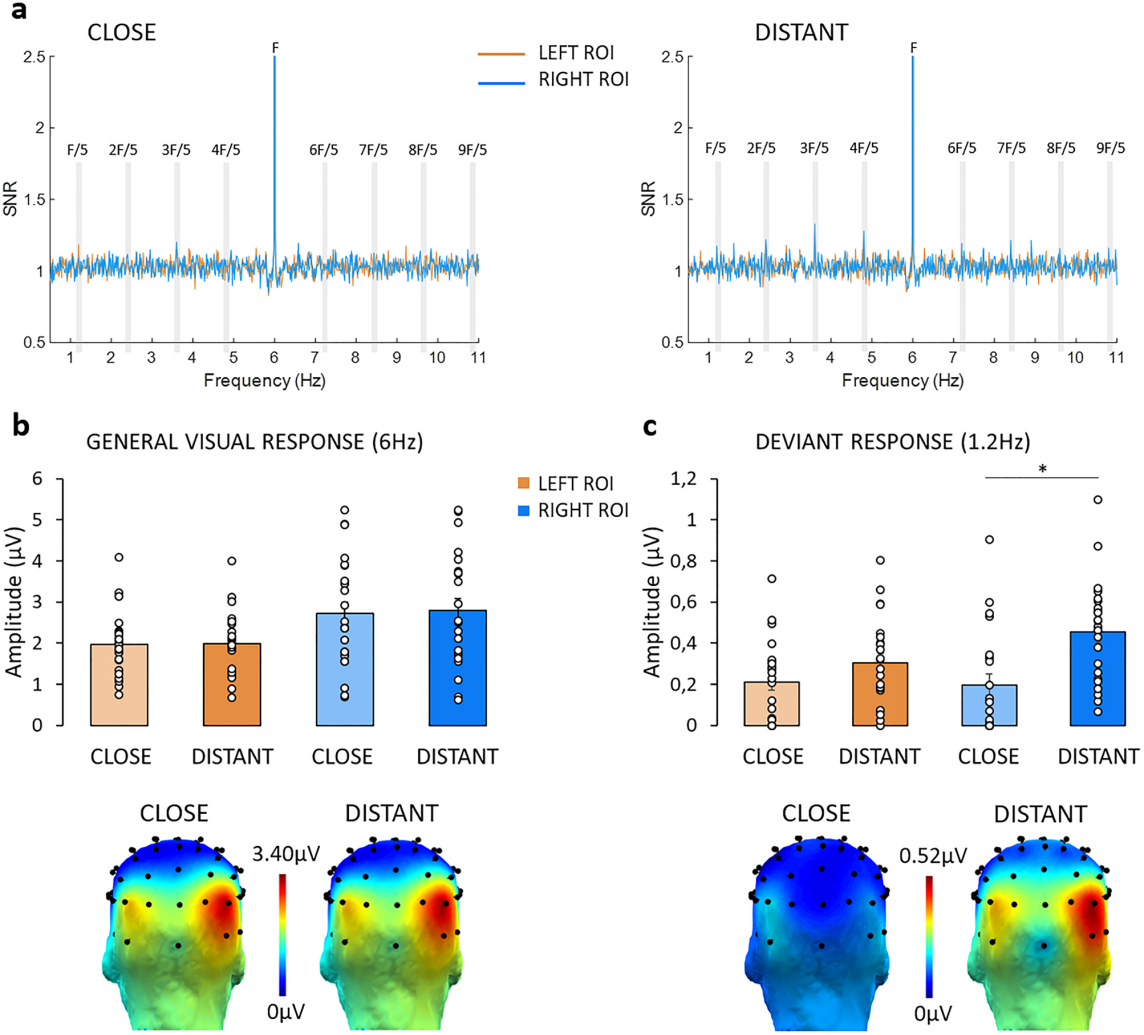
(**a**) Averaged SNR response spectra show the response peaks at the frequencies of interest in the left (orange) and right (blue) ROI, for the close (left panel) and the distant (right panel) conditions. F = 6 Hz for the general visual stimulation and F/5 = 1.2 Hz + harmonics (highlighted in grey) for the deviant stimulation. (**b**) General visual response amplitudes and topographies. Participant’s responses in the left and the right ROI for the close and distant conditions. Topographies display the sum of noise-corrected amplitudes for the significant harmonics (from 6 to 42 Hz). (**c**) Discrimination response amplitudes and topographies. Responses elicited by the presentation of the deviant number in the close and the distant conditions, in the left and right ROI. Topographies display the sum of noise-corrected amplitudes for the significant harmonics (from 1.2 to 10.8 Hz, excluding the 6-Hz frequency). Dots show individual data and bars represent standard errors of the mean. The asterisk (*) highlights a significant difference with *p* < 0.01 reflecting stronger discrimination responses for the distant condition in the right ROI only.

Clear peaks of responses were observed in signal-to-noise ratio spectra (SNR) at the general visual stimulation rate (6 Hz, Fig. 2a) and also at the discrimination frequency, albeit visually stronger when the deviant was distant than close (1.2 Hz, Fig. 2b). Significant responses (Z > 1.64, see “Methods”) were found on 7 consecutive harmonics for general visual responses (from 6 to 42 Hz) and on 9 harmonics for discrimination responses (from 1.2 to 10.8 Hz, but excluding the 6-Hz frequency). In line with previous FPVS studies on numerical representation^31,32,38^, we expected posterior general and discrimination responses, although more visual activation was anticipated for the response to the general visual stimulation than for the discrimination process. This was indeed confirmed by a ranking of the electrodes based on their response amplitude (see “Methods”) revealing that channels P8, PO8, PO10, O2 elicited the highest general visual responses while channels P8, P10, PO8 and PO10 showed the highest discrimination responses within the right hemisphere. Those were therefore selected to create the right ROIs and contralateral channels (i.e., P7, PO7, PO9, O1 for the general response and P7, P9, PO7, PO9 for the discrimination response) were averaged to create the left ROIs. Amplitude values were then analyzed with 2 (Condition: close or distant) × 2 (Lateralization: left or right) repeated-measures ANOVAs, both for general visual responses and discrimination responses. Greenhouse–Geisser correction was applied when the assumption of sphericity was violated.

### General visual responses

Close or distant conditions did not affect general visual responses, *F*(1, 22) = 2.72, *p* = 0.11, while there was a main effect of *Lateralization*, *F*(1, 22) = 13.55, *p* = 0.001, *ƞ*_p_^2^ = 0.38, responses being larger in the right ROI (*M* ± *SD* = 2.75 μV ± 1.36) than in the left ROI (*M* ± *SD* = 1.98 μV ± 0.77). The interaction between *Condition* and *Lateralization* was not significant, *F*(1, 22) = 1.43, *p* = 0.24 (Fig. 2b).

### Discrimination responses

#### Distance effect

Results revealed a main effect of *Condition*, *F*(1, 22) = 10.45, *p* = 0.004, *ƞ*_p_^2^ = 0.32, with larger responses when the deviant numerosity was distant (*M* ± *SD* = 0.38 μV ± 0.17) than close (*M* ± *SD* = 0.21 μV ± 0.19) from the base numerosity (Fig. 2c). There was no effect of *Lateralization*, *F*(1, 22) = 2.73, *p* = 0.11, but a significant interaction between *Condition* and *Lateralization*, *F*(1, 22) = 4.76, *p* = 0.04, *ƞ*_p_^2^ = 0.18. Paired t-tests per hemisphere showed a clear distance effect in the right ROI (0.26 µV stronger in distant sequences; *t*(22) = − 3.99, *p* = 0.001), but not in the left ROI (0.9 µV; *t*(22) = − 1.38, *p* = 0.18) (Fig. 2c).

#### Individual sequence effect

In order to control for potential stimulus-driven low-level perceptual features that could influence discrimination of the deviant (e.g., larger discrimination response in the sequence 2/3 than 8/9 because 2 and 3 are visually more discriminable than 8 and 9?), we contrasted response amplitudes (averaged over left and right ROIs as there was no main effect of lateralization, see Fig. 2c) of individual sequence within each condition (Table 1). No difference emerged, neither in the close, *F*(3, 66) = 1.10, *p* = 0.35, nor in the distant condition, *F*(3, 66) = 1.80, *p* = 0.16, comforting us in the lack of a major confound that would be due to the stimuli themselves (the same results were obtained when analyzing responses in the left and right ROI separately, see “Supplementary Information”).

**Table 1:** Discrimination response amplitude (mean and corresponding standard deviation) for each individual sequence. Note that the amplitude values reported here result from a noise correction at the individual sequence level.

Sequence	Amplitude (μV)	
Base/deviant	*M*	*SD*
**Close**		
2/3	0.33	0.35
3/2	0.21	0.22
8/9	0.31	0.24
9/8	0.25	0.26
**Distant**		
2/8	0.48	0.38
8/2	0.34	0.26
3/9	0.54	0.42
9/3	0.34	0.31

The individual sequences analysis (Table 1) also showed that larger discrimination responses were systematically elicited when base and deviant stimuli were in ascending order (e.g., base-2 and deviant-8) than in descending order (e.g., base-8 and deviant-2). This amplitude variation due to order, however, did not compromise the distance effect, clearly present in both ascending and descending sequences (see “Supplementary Information”).

### Behavioral results

To combine speed and error measures, inverse efficiency scores (IES, RT/ACC) were computed for close and distant conditions, averaging all code pairs (Fig. 1b), and were ln-transformed to be compared. Results showed a significant distance effect, *t*(22) = 9.41, *p* < 0.001. As expected from the literature^10–12^, participants were better to discriminate pairs of distant numerosities (*M* ± *SD* = 845.92 ms ± 123.15) than pairs of close numerosities (*M* ± *SD* = 1025.78 ms ± 194.90).

**Figure 3:**
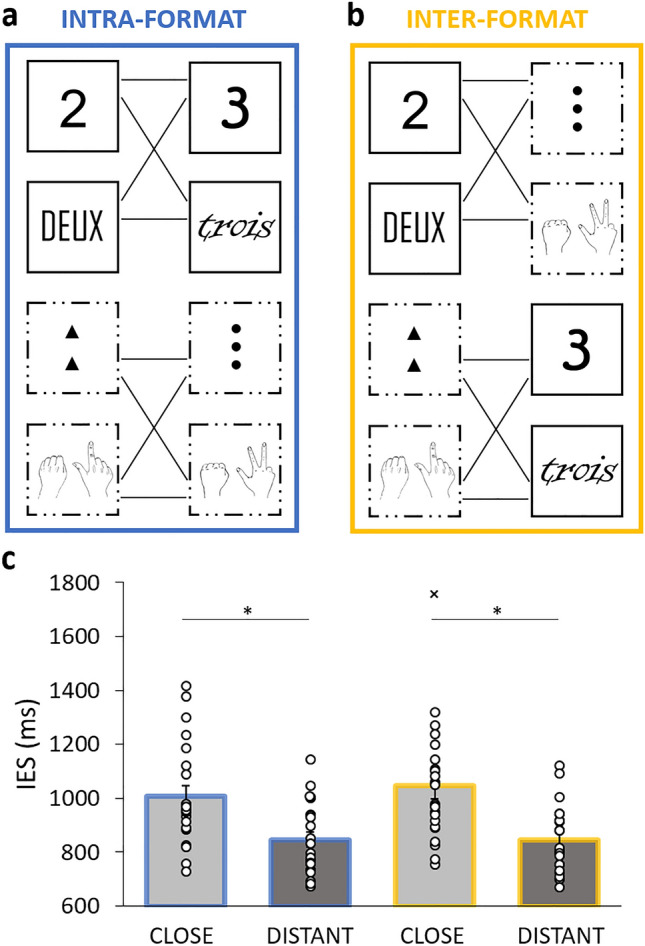
Example of pairs presented in the behavioral task. In intra-format presentations (**a**), pairs were both symbolic (i.e., digits or words) or iconic (i.e., dots or fingers); In inter-format presentations (**b**), symbolic format (i.e., digits or words) faced an iconic format (i.e., dots or fingers) and vice versa. (**c**) Participant’s mean IES in the close (light grey) and distant (dark grey) conditions, for the intra-format (blue) and inter-format (yellow) presentation types, which did not affect the distance effect. Dots show individual data and bars represent standard errors of the mean. Asterisks (*) highlight a significant difference between conditions with *p* < 0.01. The cross (˟) identifies the potential outlier.

Given that multi-code pairs included two different types of format comparisons, either same format pairs (i.e., symbolic *vs.* symbolic, and iconic *vs.* iconic, Fig. 3a “intra-format” presentations) or inter-format pairs (i.e., symbolic *vs.* iconic, Fig. 3b “inter-format” presentations), and that this factor has been debated as affecting the distance effect^13,39^, we assessed whether there was a difference between these two types of format pairs. The 2 (condition: close or distant) × 2 (format type: intra-format or inter-format) repeated-measures ANOVA revealed a main effect of *Condition*, *F*(1, 22) = 89.94, *p* < 0.001, *ƞ*_p_^2^ = 0.80, but no main effect of *Format Type*, *F*(1, 22) = 1.04, *p* = 0.32, and no interaction between *Condition* and *Format Type*, *F*(1, 22) = 1.48, *p* = 0.24. The distance effect was therefore similar regardless of whether the comparisons involved intra- or inter-format presentations (Fig. 3c). Let us note that the results of the inter-format type remained the same when removing a potential outlier (particularly high IES in the close condition of the inter-format presentations, see Fig. 3c), with a significant difference between *Conditions, F*(1, 21) = 117.14 *p* < 0.001, *ƞ*_p_^2^ = 0.85, and no other significant effects (*ps* > 0.35).

### Brain-behavior correlations

Subtraction scores were computed for each participant to quantify the distance effect both at the behavioral and brain levels, in order to relate them to each other. Behaviorally, IES in the distant condition was subtracted from IES in the close condition so that a large distance effect reflected a large difference in performance between close and distant pairs of numerosities. Distance effects ranged from 7 to 263 ms, apart from one participant who showed an abnormally high score (610 ms) and was therefore considered as a potential outlier. At the brain level, response amplitude observed in the close condition was subtracted from that in the distant condition. Only responses in the right ROI were used since the difference between conditions was not significant in the left ROI (Fig. 2c). A large brain distance effect reflected a large difference between the response amplitudes in the close and distant conditions in the right ROI.

**Figure 4:**
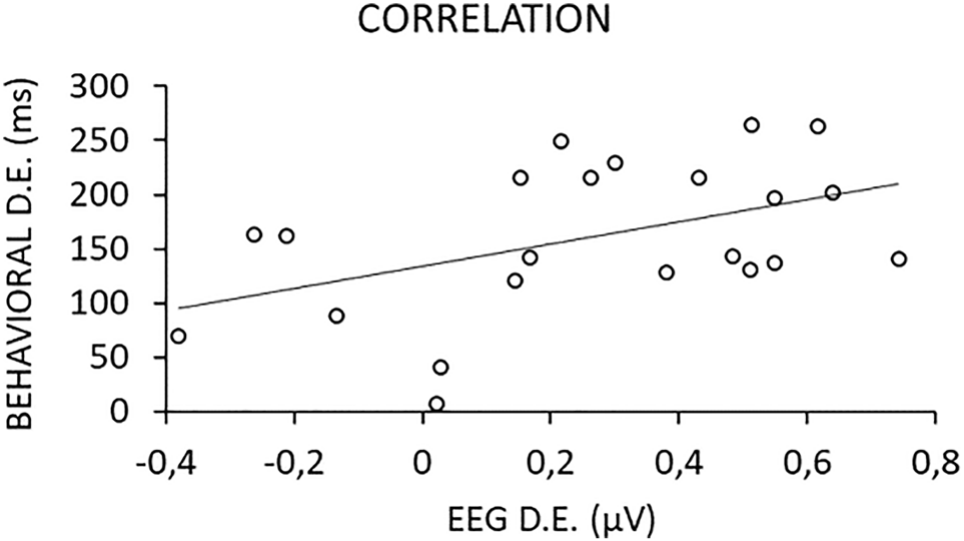
Significant correlation between the behavioral and brain distance effect (D.E.) for each participant except the outlier.

Results showed a clear relationship between participants’ behavioral and brain distance effects, revealed by a positive correlation (one-tailed Spearman), *r*_*s*_(20) = 0.426, *p* = 0.024 without the outlier, *r*_*s*_(21) = 0.387, *p* = 0.034 with the outlier’s data (Fig. 4). A greater behavioral distance effect was therefore associated with a greater brain distance effect. This link was moreover specific to discrimination responses, as no such a correlation was found (*r*_*s*_(21) = 0.051, *p* = 0.41) with the distance effect computed on the general visual response (i.e., difference between the response amplitudes at the general visual frequency, 6 Hz, in the close and distant conditions in the right ROI).

## Discussion

Although humans can deal with various representations of numerical information (e.g., set of items, fingers configurations, Arabic digits, number words), it remains a challenging question to understand if and how the brain abstracts and integrates the common underlying magnitude information. Previous studies that have attempted to address this issue have systematically been hampered by the inherent confounds between numerical magnitude and low-level physical parameters (e.g., size, density, length, shape, etc.). Here we overcame this difficulty by combining a *FPVS paradigm*—which can provide an objective and sensitive measure of numerical processing^28^—with the *distance effect*—considered as a reliable marker of numerical semantic access^16^—and a *multi-code presentation* of numerosities involving Arabic digits, number words, canonical dot patterns and canonical finger configurations. We observed significant discrimination responses of the deviant numerosity, modulated by distance, and with a topography compatible with bilateral parieto-occipital sites. Crucially, these responses reflect the brain’s ability to generalize over the deviant’s various representation codes and therefore provide robust evidence for an integration between the symbolic (i.e., digits and number words) and the iconic (i.e., canonical finger and dot patterns) formats.

Our results are in line with those of a recent event-related potential (ERP) study which showed evidence for an implicit (i.e., no decisional aspect involved) integration between the symbolic and the non-symbolic formats^16^. Greater N1 and P2p amplitudes were indeed found when participants were simultaneously presented with a set of dots and a double-digit that matched in magnitude, than when the two numerosities mismatched. In our study, access to the common magnitude information underlying the symbolic and the iconic formats was successfully assessed by the distance effect. The discrimination responses were indeed found to be modulated by the numerical distance between the deviant and the base numerosities, with a significantly stronger response when the base and the deviant were distant (i.e., from 6 units) than close (i.e., from 1 unit). However, and in line with previous ERP and fMRI studies^24,40,41^, only the right-lateralized responses were found to be modulated by the distance effect. An earlier adaptation fMRI study showed a cross-notation (dots and digits) distance effect within the right parietal cortex^40^, but the current findings extend their results in two important ways. First, we provide evidence for a higher level of integration by adding two new numerical representations—words and fingers. Second, contrary to this previous study which contrasted very large distances (i.e., 1–3 units in the close and 31–33 units in the distant conditions) that might have triggered some attentional-arousal system and amplified the activation of parietal cortex^40^, we show a more precise neural marker for distance using smaller numerosities and smaller distance between them.

Critically, the amplitude modulation due to distance was not found on the general visual responses, which confirms that the distance effect was specifically induced by the presentation of the deviant numerosity. In support to the claim that the discrimination responses reflect a reliable measure of numerical semantic access beyond mere physical aspects of the stimuli, we also showed that the individual pairs of numerosity presented (e.g., 2/3 and 8/9) did not affect the amplitude of the discrimination responses within each distance’s condition (i.e., close or distant). This reinforces the idea that the methodological choice of using a multi-code design is a promising way to overcome the impact of non-numerical factors in the evaluation of magnitude processing. Because we maximized the changes in the physical parameters of the stimuli, it is also unlikely that the brain created temporary associations or detected some regular repetitions that would have induced the discrimination responses. The fact that individual sequences did not affect response amplitudes allows us, moreover, to exclude the possibility that the use of numerosities within (i.e., 2 and 3) or outside (i.e., 8 and 9) the subitizing range^33,34^ could have influenced the results.

At the behavioral level, our data successfully showed the presence of a distance effect in the multi-code comparison task, which was reflected by better performance when participants compared distant than close pairs of numerosities. Furthermore, no cognitive cost was found when the comparisons required to switch between the numerical formats (i.e., from symbolic to iconic or vice versa), contrasting with the results of previous studies that used the non-symbolic format^13,39^. Since the iconic format leads to greater precision in the representation of numerosity than the non-symbolic format, it could also benefit from a better integration with the symbolic format. Participants indeed showed similar performance when they had to compare intra-format (i.e., symbolic vs. symbolic and iconic vs. iconic) and inter-format pairs of numerosities (i.e., symbolic vs. iconic). As in the EEG experiment, these results therefore suggest an effortless integration between the symbolic and the iconic formats. Participants’ behavioral distance effect (i.e., difference in performance between close and distant pairs of numerosities) was moreover associated with their brain distance effect (i.e., difference between the responses elicited by the presentation of a distant and a close deviant numerosity in the right hemisphere). Therefore and remarkably, these two independent measures of sensitivity to distance between numerosities were associated at the individual level, so that a greater sensitivity to distance at the behavioral level was reflected also in a greater sensitivity in brain responses. Crucially, this link between participants’ behavioral and EEG data was not found at the general visual stimulation rate (6 Hz), confirming its specificity to distance processing.

Overall, these findings support the claim that the current multi-code FPVS approach can provide an objective, sensitive and yet specific measure of magnitude processing across digits, words, dots and fingers. One limitation of the current study worth mentioning, however, concerns the *spontaneous* nature of the sensitivity to distance. Indeed, although participants were not required to do any task during the EEG stimulation sequence, they were instructed to focus on the presented numerosities so that they could tell (if asked) which was the smaller/larger at the end of a sequence. Their attention was thus pre-directed towards magnitude information and this may have contributed to the emergence of the distance effect. Future studies could therefore specifically address this question by removing any instruction or by orienting participant’s attention towards other numerical aspects, such as parity, to see whether the distance effect spontaneously emerges, or not, in these conditions.

To conclude, the current study provides strong evidence for the brain’s ability to abstract, generalize and integrate magnitude information across Arabic digits, number words, canonical fingers and canonical dots patterns. Numerical semantic access was reliably assessed by the presence of a distance effect both in the EEG experiment, which was reflected by a modulation of response amplitude according to the distance between the base and the deviant numerosities, and in an independent behavioral numerical comparison task. Critically, brain and behavioral measures of the distance effect related to each other at the individual’s level. Finally, the lack of difference in responses to individual sequences indicates that the present multi-code design, combined with a FPVS paradigm, can provide a specific, bias-minimizing measure of magnitude processing.

## Methods

### Participants

Twenty-five French speaking participants aged between 18 and 31 years old and with corrected-to-normal vision were tested, but two were excluded due to poor quality EEG signal. Analyses were therefore conducted on the remaining 23 participants (15 females, *M*_age_ ± *SD*_age_ = 22.17 ± 2.8). The procedures were in line with the Declaration of Helsinki and approved by the local “Comité d’Éthique Hospitalo-Facultaire Saint-Luc—UCLouvain” (2019/12SEP/400—B403201941534). Participants gave their written informed consent and were compensated 10 € per testing hour.

### Stimuli

Numerosities 2, 3, 8 and 9 were presented in four different codes—Arabic digit, number word, canonical dot pattern and canonical finger configuration (Supplementary Fig. S1). For each numerosity, Arabic digits and number words were presented in six different fonts. Half of the words were written in upper-case, half in lower-case letters. Variations of font and case added some variability in the low-level perceptual features. In the same vein, three different geometrical forms (i.e., circles, triangles and rectangles) were used for the canonical dot patterns. As in previous studies^38,42^, a first set of dots controlled for the density and the size of the dots while a second set controlled for the luminance and the total area occupied by the dots. Finally, canonical finger configurations were represented by the typical Belgian finger-counting configurations. Three different drawing designs were used and their mirror images were also included in the stimuli set. In total, all codes included six different representations of each numerosity (Fig. S1).

### EEG procedure

Participants were seated at 1-m distance from a screen displaying stimuli with an 800 × 600-pixel resolution. The task consisted in eight sequences of 60 s stimulation, with additional 2 s of gradual fade-in and 1 s of gradual fade-out, created and executed with a software running on JavaScript (Java SE Version 8, Oracle Corporation, USA). During the sequences, 236 × 236-pixel numerosities were visually presented at the center of the screen at a constant frequency rate of 6 Hz (i.e., 6 items per second) by means of sinusoidal modulation of contrast from 0 to 100% (Fig. 1a). One base numerosity was presented throughout the sequence and a deviant numerosity was periodically inserted every fifth item (thus at 6 Hz/5 = 1.2 Hz frequency), both presented under different codes. The stimuli were randomly chosen in the stimuli set (Fig. S1) with no immediate repetition of the same stimulus. The eight sequences were separated in four *close* pairs of base/deviant presentation (i.e., 2/3, 3/2, 8/9, 9/8) and four *distant* pairs (i.e., 2/8, 8/2, 3/9, 9/3, Fig. 1a). Participants were instructed to fixate the center of the screen and pay attention to the numerical quantities presented. They could be asked, at the end of a sequence, which was the smallest or largest number they saw. When done so (37.5% of the sequences) the task was flawless (100% accuracy). A short break was made between each sequence to ensure low artifacts in the EEG signal.

### EEG acquisition and preprocessing

EEG was recorded at 2048 Hz with a 68-channel BioSemi Active II system (BioSemi, The Netherlands). Sixty-four electrodes were placed according to the standard 10–20 system and four extra channels were added on posterior sites (PO9, PO10, I1, I2). The magnitude of the offset of all electrodes, referenced to the common mode, was held below 50 mV. Analyses of the EEG data were carried out using Letswave 6 (https://www.letswave.org), an open-source toolbox running on MatLab (The MathWorks, USA). To reduce data processing time, data files were first downsampled to 512 Hz. Then, two Fast Fourier Transform filters were applied: a band-pass with cut-off values of 0.1–100 Hz and a multi-notch at three harmonics of 50 Hz. Data were segmented 2 s before and 1 s after each sequence for inspection of artifact-ridden or noisy electrodes. Less than 2% of the channels were interpolated using the three nearest neighboring electrodes. The EEG signal was not corrected for the presence of ocular movements since the FPVS approach is highly immune to these ocular artefacts^43,44^. Each sequence was re-segmented from the stimulation onset (excluding the 2 s of gradual fade in) until 60 s, to include the largest amount of integer presentation cycles (72 cycles of 833.33 ms at the 1.2-Hz frequency). All channels were finally re-referenced to the common average.

### EEG frequency domain analyses

To allow frequency domain analyses, a Fast Fourier Transformation was applied on each sequence. Normalized amplitude spectra were obtained for the 68 channels with a frequency resolution of 0.0167 Hz (1/60 s) and three indices were extracted from the data.

First, Z-scores were computed on the average amplitude spectrum for each condition (i.e., 4 sequences in the close condition and 4 sequences in the distant) to assess significant responses at the frequencies of interest (i.e., 6 Hz for the general visual stimulation and 1.2 Hz for the deviant presentation) and harmonics. At every channel, Z-scores were calculated with the formula: $Z(x)= \frac{x - mean\left(noise\right)}{standard\;deviation(noise)}$, where *x* represents a frequency bin and the *noise* is defined as the 20 surrounding bins of each target bin excluding the immediately adjacent and the extreme (min and max) bins^45–47^. The largest chain of consecutive harmonics with a Z-score larger than 1.64 (*p* < 0.05, one-tailed, signal > noise) determined the number of significant harmonics.

Then, the amplitude at each frequency bin was divided by the noise to compute the *signal-to-noise ratio* (SNR). SNR of the four sequences per condition (close and distant) across all participants were averaged in order to visualize the general peak responses and their distribution over the significant harmonics of the frequencies of interest.

Finally, to quantify the responses and visualize their topography, sums of *noise-corrected amplitudes*—obtained by subtracting the noise from the signal of each frequency bin—were calculated on the significant harmonics of the general visual stimulation frequency (6 Hz) and the deviant discrimination frequency (1.2 Hz, but excluding the 6-Hz frequency) in each distance’s condition.

### Behavioral comparison task

The task was created and executed with E-Prime 2.0 (Psychology Software Tools, USA). The participants were seated in front of a computer screen with a response button located on either hemispace. A fixation cross first appeared on the center of the screen for 500 ms. Then two numerosities (250 × 250-pixels size) were simultaneously presented on the left and right sides of the fixation cross (at equidistance from the central cross and the edge of the screen). The participants were asked to press as fast and as accurately as possible the button on the side corresponding to the smallest or to the largest magnitude. The instruction (i.e., press for the smallest/largest) was counterbalanced between subjects. The next trial began at button press. Reaction times and accuracy were recorded for every trial.

Half the trials presented *close* pairs of numerosities (i.e., 2/3 and 8/9) and the other half *distant* pairs (i.e., 2/8 and 3/9). For each pair, numerosities were presented on the left or on the right of a fixation cross (e.g., 2-left/3-right and 3-left/2-right). In both conditions (close and distant), the four pairs of numerosities (e.g., 2/3, 3/2, 8/9, 9/8 for the close) presented each possible code (i.e., digit, word, dots or fingers) facing all the codes. For each code, the stimuli were randomly chosen within the stimuli set (Fig. S1). In total, the multi-code task consisted in 2 conditions × 4 pairs × 4 codes presented on the left of the fixation cross × 4 codes presented on the right of the fixation cross, resulting in 128 trials. The trials were divided in two blocks separated by a short break.

## Supplementary Information

Supplementary Information.

Supplementary Video S1.

## Data Availability

The EEG and behavioral data collected during the current study have been deposited in the Dryad Digital repository, https://doi.org/10.5061/dryad.612jm6469.
