# Supplementary material for: A shared numerical magnitude representation evidenced by the distance effect in frequency-tagging EEG
Source: Sci Rep. 2022 Aug 26;12:14559. doi: 10.1038/s41598-022-18811-7 (PMC9418351; doi:10.1038/s41598-022-18811-7)
Supplement: Supplementary file 1 — Supplementary Information. [file 41598_2022_18811_MOESM1_ESM.docx]

**Supplementary Information for**

A shared numerical magnitude representation evidenced by the distance effect in frequency-tagging EEG

Cathy Marlair^1,*^, Virginie Crollen^1^, & Aliette Lochy^1,2^

1. Institute of Psychology (IPSY) and Institute of Neuroscience (IoNS), Université Catholique de Louvain, Place Cardinal Mercier 10, 1348 Louvain-la-Neuve, Belgium;
2. Department of Behavioral and Cognitive Sciences, Faculty of Humanities, Social and Educational Sciences, Institute of Cognitive Science and Assessment, Université du Luxembourg, Esch-sur-Alzette, Luxembourg.

*corresponding author, e-mail: [cathy.marlair@uclouvain.be](mailto:cathy.marlair@uclouvain.be)

**Supplementary Data**

**Individual sequences, Analyzes in the left and right ROI separately.** When contrasting right hemispheric response amplitudes of individual sequences within the close and distant conditions, we found no difference, neither in the close, *F*(3, 66) = 0.56, *p* = .64, nor in the distant condition, *F*(3, 66) = 1.24, *p* = .30. The same results were obtained in the left ROI, with *F*(3, 66) = 1.32, *p* = .28 in the close condition, and *F*(3, 66) = 1.73, *p* = .17 in the distant condition.

Looking at individual sequences (table 1), it appears clearly that ascending sequences (i.e., the smallest number of the pair is the base and the largest is the deviant stimulus: 2/3, 8/9, 2/8, 3/9) systematically elicited larger discrimination responses than descending sequences (i.e., the largest number of the pair is the base and the smallest is the deviant stimulus: 3/2, 9/8, 8/2, 9/3). Averaging ascending and descending sequences within each condition, we tested for an effect of order (ascending or descending) and condition (close or distant) in a 2x2 repeated-measures ANOVA; Indeed, the main effect of Order was significant, *F*(1, 22) = 8.15, *p* = .009, *ƞ_p_*² = 0.27, ascending sequences elicited larger responses than descending ones (M_ascending_ ± SD_ascending_ = 0.41 μV ± 0.17; M_descending_ ± SD_descending_ = 0.28 μV ± 0.15). However, this effect did not interact with Condition (close/distant), *F*(1, 22) = 0.62, *p* = .44, meaning that the distance effect was present similarly for both ascending and descending sequences. A modulation of performance for ascending/descending sequences was already found in other studies before and interpreted as due to a higher frequency of occurrence in daily-life and stronger associations for ascending sequences (e.g., forward counting is more frequent than backward counting) [S1, S2]. Nevertheless, and consistently with a recent fMRI-adaptation study [S3], order did not influence the distance modulation of the discrimination responses.

S1. Vos, H., Sasanguie, D., Gevers, W., & Reynvoet, B. The role of general and number-specific order processing in adults’ arithmetic performance. *J. Cogn. Psychol.* **29**, 469–482 (2017).

S2. Wong, B., Bull, R., Ansari, D., Watson, D. M., & Liem, G. A. D. Order processing of number symbols is influenced by direction, but not format. *Q. J. Exp. Psychol*. **75**, 98–117 (2021)

S3. Goffin, C., Vogel, S. E., Slipenkyj, M., & Ansari, D. A comes before B, like 1 comes before 2. Is the parietal cortex sensitive to ordinal relationships in both numbers and letters? An fMRI‐adaptation study. *Hum. Brain. Mapp*. **41**, 1591–1610 (2020).


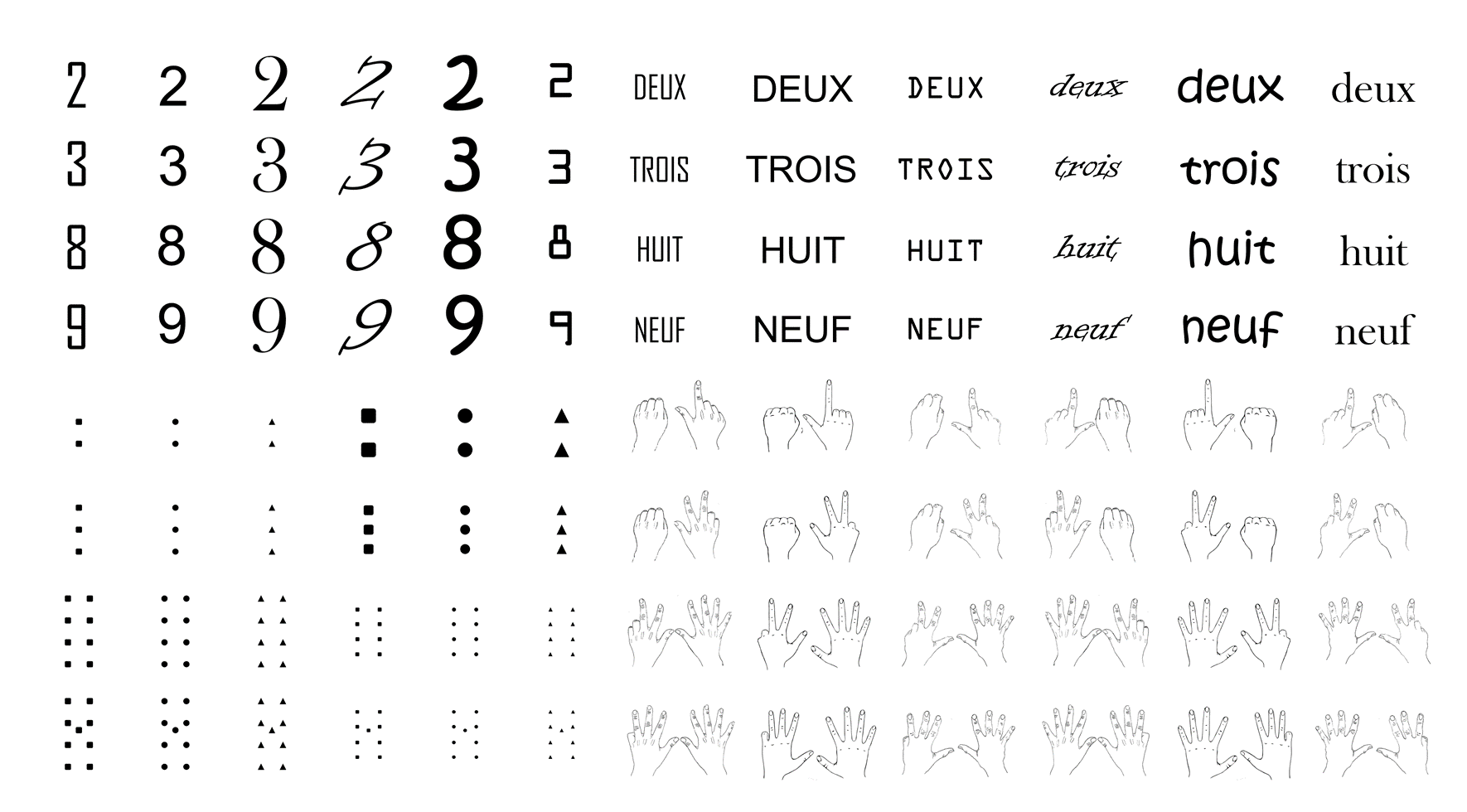


Supplementary Figure S1. Stimuli. Arabic digits, number words, canonical dot configurations (first three on the left control for the density and size of the dots, last three on the right control for the luminance and total area occupied by the dots) and canonical finger configurations.

Supplementary Movie S1. Fifteen seconds excerpt of one close (2/3) and one distant (2/8) stimulation sequence, showing numerosities presented in various codes (i.e., digits, words, dots, fingers) at 6 Hz, with the deviant numerosity (i.e., 3 or 8) appearing every five items (i.e., 1.2 Hz).
